# How to be a good partner and father? The role of adult males in pair bond maintenance and parental care in Javan gibbons

**DOI:** 10.1098/rspb.2023.0950

**Published:** 2023-06-28

**Authors:** Yoonjung Yi, Ani Mardiastuti, Jae C. Choe

**Affiliations:** ^1^ Laboratory of Animal Behaviour and Conservation, College of Biology and the Environment, Nanjing Forestry University, Nanjing, 210037, People's Republic of China; ^2^ Department of Forest Resources Conservation and Ecotourism, IPB University, Bogor, 16680, Indonesia; ^3^ Laboratory of Behavioral Ecology, Division of EcoScience, Ewha Womans University, Seoul, 03760, Republic of Korea

**Keywords:** pair bond, parental care, grooming, social playing, gibbon, grooming equality index

## Abstract

In pair-living species, female and male pairs may maintain stable social bonds by adjusting spatial and social associations. Nevertheless, each sex invests differently to maintain the pair bond, and the investment can depend on the presence of paternal care or ‘male services.’ While most species live in pairs, the sex responsible for pair bond maintenance in gibbons is still controversial. We investigated pair bond maintenance and parental care in three pairs of wild Javan gibbons in Gunung Halimun-Salak National Park, Indonesia, for over 21 months. We found that Javan gibbon fathers groomed their offspring more than adult females, especially as offspring got older. While both parents increased playing time with offspring when offspring became older and more independent, fathers played with offspring 20 times more than mothers on average. Grooming within Javan gibbon pairs was male-biased, suggesting that pair bond maintenance was heavily the job of males. However, offspring age as a proxy for paternal care did not affect the pair bond maintenance. Our study highlights that adult male Javan gibbons may have an important role in pair bond maintenance and the care of juveniles.

## 1. Introduction

Pair bonds between two adult individuals are attachment relationships that can benefit both individuals by securing food sources, jointly defending territory, mating and siring offspring and reducing stress (reviewed in [1]), even though in some species, pairs may not perform all of the list above [2–4]. Generally, pair-living females and males maintain stable social bonds by adjusting spatial and social associations, which can be energetically demanding [5]. Nevertheless, each sex invests differently to maintain the pair bond [6]. Females may invest more to maintain the pair bond than males when males provide some services, such as direct infant care or protection of other group members from predators [7,8]. On the other hand, males may invest more to maintain the pair bond than females in order to increase mating opportunities, according to the females-as-a-limited-resource hypothesis [9,10].

Various pair-living primate males invest more than females in maintaining the pair bond (*Hoolock hoolock*: [11]; *Hylobates lar*: [9]; *Indri indri*: [12]; *Callimico goeldii*: [13]), while researchers also found different results showing that females invest more than males (*H*. *lar*: [14]; *Callicebus torquatus*: [15]; *Pithecia pithecia*; [16]; *Plecturocebus cupreus*; [17]), using grooming as a proxy for pair bond maintenance, given its important role in primate social interactions [5]. The period of paternal care or offspring development might cause inconsistent results between and within a species [18]. For instance, females were more responsible for maintaining the pair bond in red titi monkeys (*P*. *cupreus*), the species in which mostly males carry their infants. Moreover, red titi monkey females groomed males significantly more with the presence of infants, most likely because females are ‘paying back’ the males for carrying the infants [17]. Similarly, the relationship within white-faced saki (*P*. *pithecia*) pairs changed with offspring development, as adult females were more responsible for maintaining proximity with adult males in the presence of dependent offspring than independent offspring [16]. Even without direct paternal care shown in titi monkeys, white-faced saki females might value service from males (e.g. infant protection) more when the energetic cost to take care of dependent offspring is higher for females [16].

Another question arises here: How can we quantify males' services? For instance, are males providing direct or indirect paternal care? Maternal care has been extensively studied in a wide range of mammalian taxa, mainly focusing on breastfeeding or infant carrying, tasks directly related to offspring survival [19–22]. By contrast, paternal care in primates has been understudied even though paternal care is more common in primates than in other mammalian orders [23–26], and primate fathers provide diverse parental care such as carrying, food transfer, playing, grooming, protection against infanticide and support in aggressive interactions [7,27–32]. Furthermore, following the definition of relevant studies, direct paternal care includes the first four and indirect care includes the latter two, despite some discordant definitions depending on the studies [26,33,34]. Moreover, some paternal care is concentrated during infancy, but grooming or playing increases when offspring become juveniles [27,35], while maternal care decreases [36]. Juvenile periods in primates are prolonged compared to other mammals, despite the increased risks linked to increased mortality during this life stage [37,38]. Therefore, paternal care can play an important role in helping juveniles acquire adult-level social skills that can compensate for delayed maturation costs [39]. Juveniles receive less extensive maternal care after weaning [40,41], emphasizing fathers' role in pair-living primates that lack kin or peers.

Gibbons have the third-longest juvenile period among the 27 species of primates analysed in the cited work, accounting for 22.2% of their lifespan, following common woolly monkeys (*Lagothrix lagotricha*) with 60.9% and humans (*Homo sapiens*) with 24.2% [37]. The prolonged juvenile period of gibbons most likely indicates the need for immatures to acquire ecological and social knowledge before becoming adults. However, except for some indirect forms of caregiving (e.g. defense against predators or infanticide [42–44]) and direct care from siamang males (*Symphalangus syndactylus*) carrying infants in their second year of life [45], studies on the gibbon father–offspring relationship are generally lacking [46]. Further, research on sex-biased investment in gibbons draws controversial results between and within species (reviewed in [18]), indicating a need to investigate family dynamics concerning sex-biased investment in maintaining the pair bond and providing parental care.

Javan gibbons (*Hylobates moloch*) are strictly pair-bonding compared to other gibbon species with more flexible social structures [44,47–49]. In this study, we investigated the family dynamics of wild Javan gibbons at Gunung Halimun-Salak National Park, Indonesia. First, we examined maternal and paternal care differences with offspring age to investigate each sex's parental effort (i.e. breastfeeding, carrying, grooming and playing). We predicted a positive relationship between paternal effort and offspring age and a negative relationship between maternal effort and offspring age. Then, we investigated the sex responsible for pair bond maintenance and how the contribution changes with offspring age as a proxy for parental effort (e.g. male services). We predicted that males would invest more (i.e. groom females more than *vice versa*) than females in general, following the females-as-a-limited-resource hypothesis due to the lack of direct paternal care in Javan gibbons. We also predicted that the males' investment in the pair bond would decrease as the infant gets older due to increased demands for paternal care in the juvenile period. Since ecological factors such as food availability or temperature also might affect how much gibbons can afford these two energy-costly behaviours (i.e. pair bond and parental care), we investigated the questions above while controlling for these ecological factors.

## 2. Material and methods

### (a) Study site and subjects

The field site is in the Citalahab area of the Gunung Halimun-Salak National Park (6°44′S, 106°31′E) in West Java, Indonesia. We collected data from three habituated wild Javan gibbon groups A, B and S. All three groups consist of an adult female–male pair and their offspring (total *N* = 12). We followed the age classification from Brockelman *et al.* [50] (infant, 0–2 years; juvenile, 2–5 years; adolescent, 5–8 years; subadult, 8 years–dispersal). Groups A and B had two offspring each throughout the study. Group S had three offspring and we excluded the oldest offspring from the data analysis since the individual dispersed in April 2016, near the end of the study.

### (b) Data collection

We carried out all-day field observations on the three habituated gibbon groups from one sleeping tree to the next over 21 months between November 2014 and July 2016. We observed them for 2209 h over 306 days (group A: 776.25 h over 105 days; B: 720.5 h over 101 days; S: 712.25 h over 100 days). During the study period, we collected data from an adult female–male pair in each group, and a younger offspring (10–30 months) and an older offspring (45–65 months) from group A, and two younger offspring (5–25 months) and two older offspring (41–61 months) from groups B and S. We recorded the occurrence of social grooming (hereafter ‘grooming’) and social playing (hereafter ‘playing’) bouts between all family members using all occurrence sampling. We defined a grooming bout as grooming without ceasing for longer than one minute in between [52]. Particularly for the mother–offspring dyad, we recorded the time mothers breastfed and carried infants using 15 min focal sampling (all occurrence sampling during 15 min) for every hour [51].

We used grooming to represent an investment in the pair bond between an adult female and an adult male and as a proxy for parental care from parents to offspring. We used the terms ‘mother’ and ‘father’ interchangeably with ‘adult female’ and ‘adult male’ in the parent–offspring relationship, as only one adult female and one adult male were in the group. Even though we do not have data on paternity since no extra-pair copulation has been observed for 17 years of the intensive long-term research, we assume the adult males are likely genetic fathers of the offspring.

For control variables, we recorded monthly fruit availability from trees with a diameter at breast height (dbh) ≥ 10 cm and lianas with dbh ≥ 7 cm, from 25 randomly selected phenology plots (10 m × 50 m) in gibbons' home ranges (for details see [53,54]). We recorded daily maximum and minimum temperatures from an electronic temperature data logger (Model 1441, Taylor, Oak Brook, IL), which we used to calculate the daily mean temperature.

### (c) Data analysis

#### (i) Parental care: breastfeeding and carrying

To examine the changes in maternal care during infancy, we fitted two models with a beta error distribution and logit link function using R package *glmmTMB* [55]. For the first model, we included the daily proportion of breastfeeding time (breastfeeding duration/total observation time; *N* = 258) as the response variable. We included the infant age (months) as the test predictor, and fruit availability and mean temperature as the control predictors. We then included the gibbon group ID as the random factor, and all the test and control predictors as the random slope within the gibbon group ID. For the second model, we included the daily proportion of time mothers carried infants (carrying duration/total observation time; *N* = 265) as the response variable, and the identical test and control predictors, random factors and random slopes as the first model. Since fathers did not breastfeed or carry offspring of any developmental stage, we analysed mother–infant relationships only.

#### (ii) Parental care: grooming and playing

To examine the changes in parental care, we fitted two models with a beta error distribution and logit link function using R package *glmmTMB* [55]. For the first model, we included the daily proportion of parental–offspring grooming time per each parent and offspring (grooming duration/total observation time; *N* = 1072) as the response variable. We included the interaction between infant age (months) and parent ID (father or mother) as the test predictors, and fruit availability, mean temperature and gibbon group size (number of all group members) as the control factors. We then included the gibbon group ID as the random factor, and all the test and control predictors as the random slope within the gibbon group ID. For the second model, we included the daily proportion of parent–offspring playing time per each parent and offspring (playing duration/total observation time; *N* = 1044) as the response variable, and identical test and control predictors, random factors and random slopes to the first model.

#### (iii) Pair bond maintenance

To investigate which sex invests more than the other, we first calculated the grooming equality index for each pair: (1 − (*G*_fm_ − *G*_mf_)/(*G*_fm_ + *G*_mf_))/2 (modified from Silk *et al.* [56]). Here *G*_fm_ indicates the time females spent grooming males. The grooming equality index ranges from 1 (male-biased grooming) to 0 (female-biased grooming). Then we calculated the average grooming equality between male and female gibbon pairs. Afterwards we fitted a model with a beta error structure and logit link function using the R package *glmmTMB* [55], including the grooming equality index between females and males (*N* = 240) as the response variable, the infant age (months) as the test predictor, and fruit availability and mean temperature as the control predictors. We then included the gibbon group ID as the random factor, and all the test and control predictors as the random slope within the gibbon group ID. To avoid zeros and ones in the response variable, we compressed the response variable using the formula *y*′ = (*y* × (*n* − 1) + 0.5)/*n*, where *n* represents the sample size [57].

We z-transformed all quantitative predictors to a mean of 0 and standard deviation of 1 before fitting the models. All the models included theoretically identifiable random slopes for the fixed effects within random intercepts. We checked for collinearity among predictors using the package *car* [58] and found no collinearity issues. We ran likelihood ratio tests with null models that include random factors and control predictors only. We discussed the results of the model only when a full-null model comparison showed significance or a trend [59,60]. We also conducted Tukey tests to perform *post hoc* comparisons using the R package *lsmeans* [61]*.* While having at least five levels for random effect terms is generally recommended, we still include gibbon group ID (*N* = 3) as a random factor given the support from other studies suggesting two levels would still correctly estimate the variance [62,63]. All data were analysed using R (v.4.1.1; [64]).

## 3. Results

### (a) Parental care: breastfeeding and carrying

**Figure 1.**
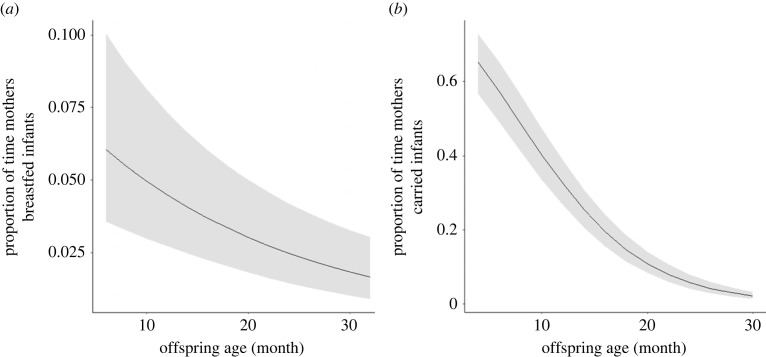
Effects of the offspring age on (*a*) the daily proportion of time mothers spent breastfeeding infants (breastfeeding duration/total observation time) and (*b*) the daily proportion of time mothers spent carrying infants (carrying duration/total observation time) in Javan gibbons in Gunung Halimun-Salak National Park between November 2014 and July 2016. The shaded areas represent the 95% confidence intervals.

The two GLMM models showed that as the infant grew, the time mothers breastfed (*B* = −0.320, s.e. = 0.066, *z* = −4.810, *p* < 0.001; electronic supplementary material, table S1; figure 1*a*) and carried the infant (*B* = −1.076, s.e. = 0.071, *z* = −15.260, *p* < 0.001; electronic supplementary material, table S2; figure 1*b*) decreased. When infants were younger than 10 months old, mothers carried infants more than 50% of the time and breastfed them around 5% of the time, but as infants grew to 25–30 months old, both breastfeeding and carrying ceased.

### (b) Parental care: grooming and playing

**Figure 2.**
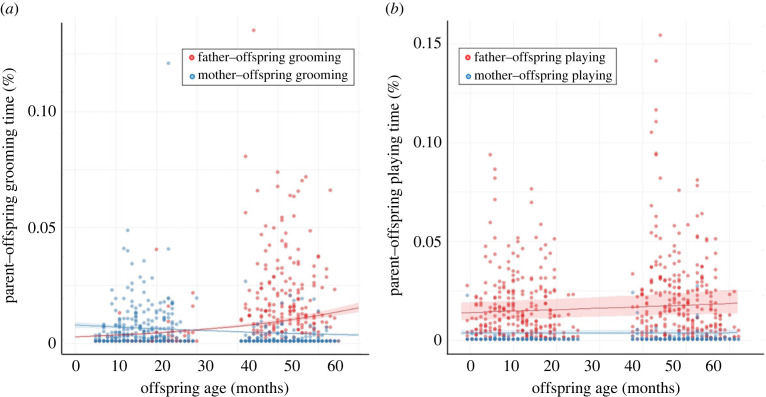
Effects of offspring age and parent sex (father or mother) on (*a*) daily proportion of parent–offspring grooming time (grooming duration/total observation time) and (*b*) daily proportion of parent–offspring playing time (playing duration/total observation time) in Gunung Halimun-Salak National Park between November 2014 and June 2016. The shaded areas represent the 95% confidence intervals.

On average, fathers groomed their offspring for 0.8 ± 1.5% of their daily activity time and mothers groomed their offspring for 0.3 ± 0.8% of their daily activity time. Moreover, fathers played with their offspring for 0.4 ± 1.5% of the time and mothers played with their offspring for 0.02 ± 0.2% of the time. The model investigating the parent–offspring grooming time revealed that the interaction between offspring age and parent sex was significant (full-null model comparison: *χ*^2^ = 22.93, d.f. = 3, *p* < 0.001). When offspring got older, fathers groomed offspring more than mothers groomed offspring (GLMM: *B* = −0.706, s.e. = 0.097, *z* = −7.247, *p* < 0.001; electronic supplementary material, table S3; *post hoc* for parent ID × offspring age: fathers versus mothers, *p* < 0.001; figure 2*a*). The result from the model investigating the parent–offspring playing time was similar (full-null model comparison: *χ*^2^ = 580.17, d.f. = 3, *p* < 0.001) in that the fathers spent much more time playing with offspring than the mothers did (GLMM: *B* = −1.457, s.e. = 0.056, *z* = −25.907, *p* < 0.001; electronic supplementary material, table S4; *post hoc* for parent ID: fathers versus mothers, *p* < 0.001) and both parents played more with offspring as the offspring got older (*B* = 0.093, s.e. = 0.030, *z* = 3.090, *p* = 0.002; electronic supplementary material, table S4; figure 2*b*).

### (c) Pair bond maintenance

**Figure 3.**
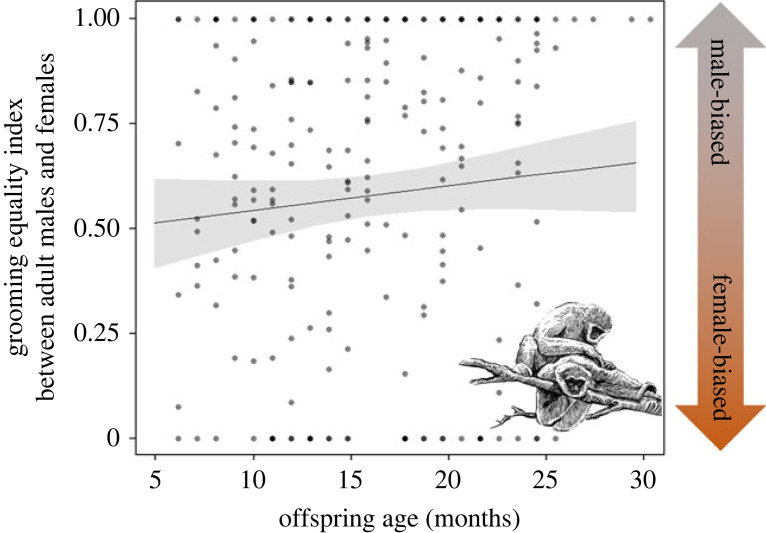
Effects of the offspring age on the grooming equality index between adult male and adult female pairs of Javan gibbons in Gunung Halimun-Salak National Park between November 2014 and July 2016. The shaded areas represent the 95% confidence intervals.

In general, adult male–adult female grooming equality index was 0.62 ± 0.36, indicating male-biased grooming. However, the full-null model comparison investigating the effect of offspring age on grooming within the pairs was not significant (*χ*^2^ = 1.940, d.f. = 1, *p* = 0.160; figure 3).

## 4. Discussion

Our study investigating pair bond maintenance and parental care in three wild Javan gibbon families highlights the social dynamics in family groups. We found that Javan gibbon parents have a distinct role in parental care, highlighting that paternal care focused on and increased during the juvenile period of offspring life. Then, grooming within Javan gibbon pairs was adult male-biased, but the grooming equality index did not change with offspring age, contrary to our prediction. Our study shows that adult male Javan gibbons may have an important role in caring for juveniles and in pair bond maintenance.

In most mammals, when infants are young and unweaned, they stay in proximity to their mothers mainly because of breastfeeding and locomotor dependence. Maternal care in infancy is crucial in gibbons especially because their high arboreality demands more complex locomotor adjustments than on the ground [65]. For example, siamang infants disappeared and most likely died when no one carried the infants, which was shortly after newly immigrated males evicted the infant's father (the main carriers in siamangs) [66]. This suggests the need for parental care in carrying during the infancy of gibbons, which would be maternal care in the case of Javan gibbons, as only mothers carry infants. Javan gibbon fathers did not carry offspring, yet they interacted more than the mother did through grooming and playing with their offspring. For example, fathers groomed their offspring more than adult females did, a pattern that increased as the offspring got older. While both parents increased playing time with offspring when offspring became older and more independent, and fathers played with offspring 20 times more than mothers did, on average. Juvenile Javan gibbons can interact with only two to three group members more experienced than themselves, and they interact mainly with fathers or older siblings as mothers primarily focus on taking care of infants. In two out of three of our study groups, juveniles did not have older siblings, making them dependent on fathers for social interactions. Outside of the grooming or playing contexts, juveniles also stayed in close proximity, co-foraged and slept together with their fathers [67]. These close relationships with fathers, including extensive grooming and playing, are essential to juveniles' welfare [68], indicating that those direct social interactions should certainly be regarded as direct care. To sum up, our study highlights that maternal care during infancy and paternal care during juvenility are the key to parental care in the Javan gibbons.

**Figure 4.**
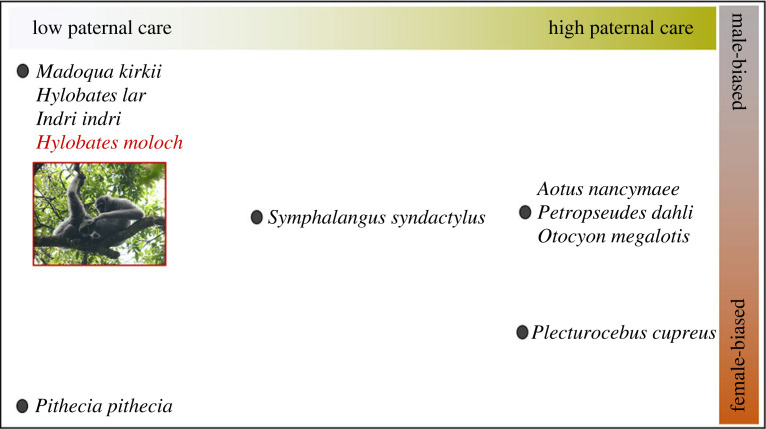
Pair-living mammal species in a frame of pair bond maintenance investment (male-biased investment for above midline, female-biased investment for below midline, and equal investment at midline) and paternal care (low paternal care for left half, high paternal care for right half, and intermediate paternal care of the middle part), modified from Dolotovskaya *et al.* [17]. Note that only data from the wild are considered (except for *Aotus nancymaee*), data from non-primate species are from approach/leave data [74–77], and data from primates are a mix of grooming reciprocity and approach/leave data [9,12,16,17,78,79]. Red font indicates the focal study subject, with a picture of a father Javan gibbon grooming a juvenile in Gunung Halimun-Salak National Park, Indonesia. © Yoonjung Yi.

On the other hand, we found that the pair bond maintenance of the Javan gibbons was heavily the job of males. Grooming between adult female and male Javan gibbons was skewed, indicating that males invested more to maintain the pair bond than females did. Male Javan gibbons do not carry offspring, defend food resources or protect group members against predation, but they defend female mates or infants from outgroup males [44,69]. While our results support male-biased investment in general, whether grooming is sex-biased or equal has been debated as many studies presented controversial results, even within Hylobatidae (reviewed in [18]). For instance, in wild *H. lar*, studies have found male-biased grooming in three pairs, female-biased grooming in two pairs and equal grooming in one pair [42,70–72], and the results are more complicated when including other genera in Hylobatidae or species in captivity [18,73]. This controversy might be because of the different social and reproductive contexts. Our results align with those of other pair-living mammal species in the sense that species with high paternal care showed more equal investment in pair bond maintenance from both sexes, and species with low paternal care showed male-biased investment (figure 4). More studies on non-primate pair-living species would clarify the relationship between pair bond maintenance and parental care.

However, the model investigating the effect of offspring age on grooming equality within pairs found that offspring age could not explain the variation of the grooming equality index, which disagrees with our prediction that it would be less male-biased considering the amount of paternal care for older offspring. A potential explanation for the result would be that grooming and playing with offspring might be less energetically costly than other intensive paternal care (e.g. carrying), which affects biased investments in titi monkeys [17]. Future studies measuring the exact cost of paternal care would help to understand the impact of the behaviour. Another factor that might affect the pair bond maintenance that has not been considered in this study is female reproductive cycling. It may be more common for males to groom females during the period when mating is more likely to occur, as grooming is often used as a commodity for mating [52,80]. For example, males groomed females more when females were in oestrus, based on hormonal analysis in *H. lar* [52]. Further studies investigating pair bond maintenance with more short-term-based methods (i.e. hormonal analysis) could confirm the impact of female cycling and mating opportunities on grooming within pairs in other gibbon species.

## Ethics

We observed the behaviours of gibbons without any invasive methods. Our research protocol was approved by the Indonesian Ministry of Research and Technology (RISTEK; permit nos. 375/SIP/FRP/SM/X/2014, 91/P/TNGHS/2015 and 652/FRP/SM/VI/2015), the Indonesian Ministry of Forestry's Department for the Protection and Conservation of Nature (PHKA), and the Gunung Halimun-Salak National Park.

## Data Availability

Data are available from the Dryad Digital Repository: https://doi.org/10.5061/dryad.6wwpzgn41 [81]. Additional information is provided in electronic supplementary material [82].
